# Thermo-Responsive Hydrogel Based on Lung Decellularized Extracellular Matrix for 3D Culture Model to Enhance Cancer Stem Cell Characteristics

**DOI:** 10.3390/molecules29184385

**Published:** 2024-09-15

**Authors:** Lei Chen, Fanglu Li, Ruobing Li, Ke Zheng, Xinyi Zhang, Huijing Ma, Kaiming Li, Lei Nie

**Affiliations:** 1 Key Laboratory of Tea Plant Biology of Henan Province, College of Life Sciences, Xinyang Normal University, Xinyang 464000, China; lifanglululu@126.com (F.L.); ruobinglizlrb@163.com (R.L.); zhengkeczk@163.com (K.Z.); cnnyzhangxy@163.com (X.Z.); likaiming@163.com (K.L.); 2 Library, Xinyang Normal University, Xinyang 464000, China; huijing_ma@163.com

**Keywords:** hydrogel, cancer stem cells, 3D cell culture, EGCG, metastasis

## Abstract

Cancer stem cells (CSCs) are most likely the main cause of lung cancer formation, metastasis, drug resistance, and genetic heterogeneity. Three-dimensional (3D) ex vivo cell culture models can facilitate stemness improvement and CSC enrichment. Considering the critical role of extracellular matrix (ECM) on CSC properties, the present study developed a thermo-responsive hydrogel using the porcine decellularized lung for 3D cell culture, and the cell-laden hydrogel culturing model was used to explore the CSC characteristics and potential utilization in CSC-specific drug evaluation. Results showed that the lung dECM hydrogel (LEH) was composed of the main ECM components and displayed excellent cellular compatibility. In addition, lung cancer cells 3D cultured in LEH displayed the overexpression of metastasis-related genes and enhanced migration properties, as compared with those in two-dimensional (2D) conditions. Notably, the CSC features, including the expression level of stemness-associated genes, colony formation capability, drug resistance, and the proportion of cancer stem-like cells (CD133^+^), were also enhanced in 3D cells. Furthermore, the attenuation effect of epigallocatechin gallate (EGCG) on CSC properties in the 3D model was observed, confirming the potential practicability of the 3D culture on CSC-targeted drug screening. Overall, our results suggest that the fabricated LEH is an effective and facile platform for 3D cell culture and CSC-specific drug evaluation.

## 1. Introduction

Lung cancer is the most prevalent malignancy and the leading cause of cancer-related death globally, with 11.4% of incidence and 18% of mortality, respectively, in 2020 [1,2]. For now, lung cancer is the most frequent cancer in men, both in cases and deaths [3]. The lung cancer treatments mainly include surgery, chemotherapy, radiation, and targeted medicines; however, the prognosis for lung cancer patients is still unsatisfactory in recent decades [4,5]. Further exploring cancer stem cell (CSC) characteristics in lung cancer could facilitate the search for lung cancer treatment and new drug development [6,7]. CSCs, a small population of cancer cells with the properties of self-renewal, migration, invasion, differentiation into heterogeneous cells, and drug resistance were suggested to be responsible for cancer progression, metastasis, and chemoradiotherapy resistance in lung cancer [8,9]. Surely, numerous studies have shown that the development of CSC-specific drugs offers the promise of improving the clinical cure of cancer [10,11]. However, despite improving knowledge about CSC properties obtained from two-dimensional (2D) conditions, the inherent deficiency of the 2D condition, which was the failed emulation of the complicated CSC microenvironment, limited its application [12]. In addition, CSCs were considered the main driving force of tumor development and progression; especially the reciprocal communication of CSCs and the microenvironment further leads to enhanced proliferation and metastatic capability. Thus, the tumor microenvironment plays an active role in tumor progression, and the maintenance of stemness in cancer cells in the tumor microenvironment could facilitate the discovery of the new targeted drug [13,14]. Although animal models are considered more similar to the microenvironment of cancer in vivo, their time-consuming nature, high cost, and genetic heterogeneity with humans hinder drug screening for lung cancer therapy [15]. Therefore, it is urgent to develop effective and cost-effective platforms for CSC investigation to overcome the limitations of 2D conditions and animal models. 

Many recent studies have demonstrated that 3D culture is an effective platform for CSC enrichment in vitro [16,17]. Indeed, cancer cell culture in the 3D condition, in the form of cell spheroids, multi-layer structures, or scaffold attachment, displayed an improvement in CSC behaviors, including enhanced invasion, drug resistance, and tumorigenesis, with activating CSC signal pathways [18,19]. The application of 3D culture for CSC enrichment and propagation provides a valuable alternate model for the development of CSC-targeted drugs with potential clinical applicability [20,21]. The 3D matrix profoundly influences cell characteristics, and the reciprocity between the matrix and cells is important for in vitro models in drug screening [22]. Emerging evidence suggests that extracellular matrix (ECM) proteins serve as a physical and biochemical niche for cell attachment to drive the stemness of cancer cells [23,24]. For example, collagens, the main structural elements of ECM protein, could efficiently improve the metastasis, self-renewal, proportion, and drug resistance of CSCs [25]. Thus, acellular tissue materials composed of ECM components, including fibrous proteins, glycosaminoglycans, proteoglycans, and sequestered growth factors, have attracted more attention in the field of 3D CSC culture [26,27]. In addition, scaffolds based on ECM have been employed in tissue regeneration, such as wound regeneration [28], peripheral nerve regeneration [29], and so on [30]. More importantly, the fabrication technology of tissue ECM hydrogel, which could efficiently improve the communication between cancer cells and ECM proteins, facilitates the evaluation of the characteristics of CSCs via the 3D cell culture [31]. 

**Scheme 1:**
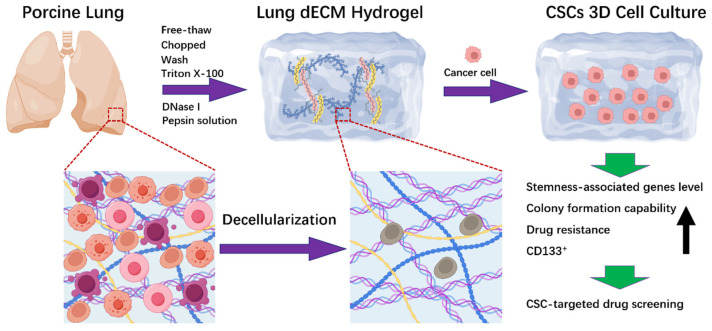
The schematic illustration of fabricating lung dECM hydrogel (LEH) using porcine lung via the decellularization process for CSCs 3D cell culture.

In the present study, the porcine lung was employed to prepare the decellularized ECM via a decellularization process, and then, the thermo-responsive ECM hydrogel was facilely fabricated. Next, the engineered lung ECM hydrogel (LEH) was used for 3D cancer cell culture to upregulate the expression of CSC characteristics (Scheme 1). The physicochemical properties of LEH, such as the microstructure and rheological properties, were investigated. The ECM components in LEH were analyzed by pathological section examination and integrated into the 3D culture model. The upregulation of CSC properties, including migration, self-renewal, drug resistance, and stem-like cell proportion, was studied in 3D conditions. More importantly, the feasibility of the 3D model utilized for CSC-specific drug screening was characterized by detecting the inhibition effect of epigallocatechin gallate (EGCG) on CSC properties. 

## 2. Results

### 2.1. Preparation and Characteristics of LEH

**Figure 1:**
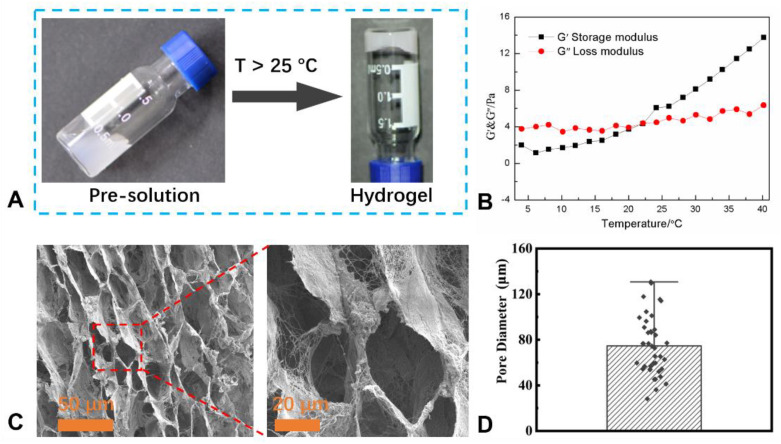
(**A**) Lung dECM hydrogel (LEH) could be fabricated using lung decellularized matrix via increasing temperature over 25 °C. (**B**) Storage modulus (G′) and loss modulus (G″) of LEH over temperature, indicating the sol–gel transition process. (**C**) SEM images of dried LEH using different magnifications show the porous microstructure. (**D**) Pore size distribution analysis of LEH based on SEM images using ImageJ software (Version 1.54).

In this work, the decellularized matrix was prepared using the porcine lung, and then, the lung dECM hydrogel (LEH) was fabricated (Scheme 1). LEH could be facilely obtained via increasing temperature over 25 °C, and the sol–gel transition process is shown in Figure 1A. The transition from the opaque liquid to white hydrogel could be investigated using a tube verting method [32,33]. In addition, a rheometer was used to further confirm the sol-to-gel transfer, and the storage modulus (G′) and loss modulus (G″) in terms of temperature was shown in Figure 1B. The gelation temperature could be verified using the crossover point of G′ and G″; before the crossover point, G″ was larger than G′, and after the point, G′ became larger than G″, indicating the gelation state formation. Furthermore, the microstructure of LEH was observed using SEM, and SEM images of LEH are displayed in Figure 1C. The SEM images showed the interconnected porous structure of LEH, and plenty of micropores were observed. According to ImageJ software (Version 1.54) analysis, the average pore size of LEH was 75 ± 25.1 μm (Figure 1D). 

**Figure 2:**
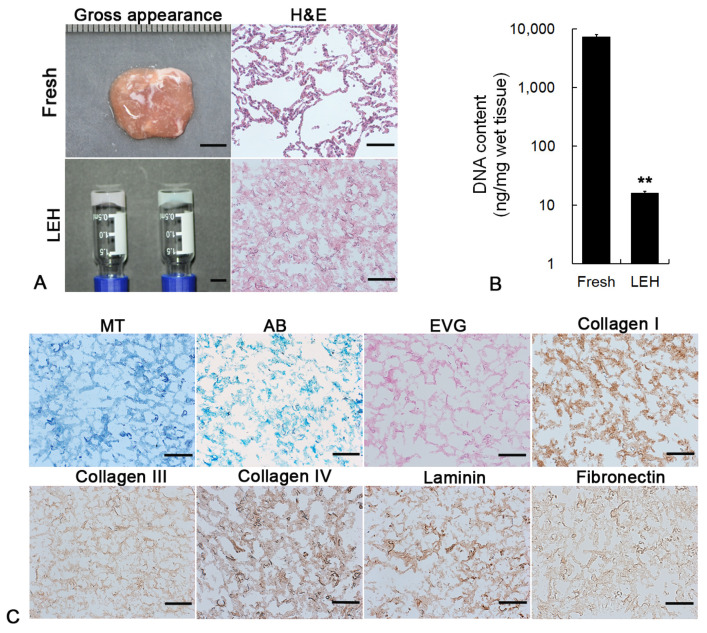
(**A**) Gross appearance and H&E staining of fresh lung tissue and LEH. Scale bar: gross appearance, 5 mm; H&E staining, 100 μm. (**B**) Quantification of DNA content in fresh lung tissue and LEH. (**C**) Histological and IHC staining of ECM components. MT, AB, and EVG staining were utilized to visualize collagen, elastin, and GAGs, respectively. IHC staining showed the retention and distribution of collagen I, collagen III, collagen IV, laminin, and fibronectin in LEH. Scale bar: 100 μm. ** *p* < 0.01.

Next, the histological and IHC staining was further used to investigate the physicochemical properties of LEH, and the quantity of residual DNA in LEH was quantified. As indicated in Figure 2A, the filament structure and the DNA removal of LEH dissolved in the medium were displayed in H&E staining. Measuring DNA content further confirmed minimal DNA preserved in hydrogel (Figure 2B). The main components of ECM, including collagens, elastin, and glycosaminoglycans, were preserved in hydrogel as indicated by MT, AB, and EVG staining (Figure 2C). IHC results revealed the retention and distribution of key ECM proteins in LEH, including collagen I, collagen III, collagen IV, laminin, and fibronectin. 

### 2.2. Establishment of 3D Cell Culture in LEH

**Figure 3:**
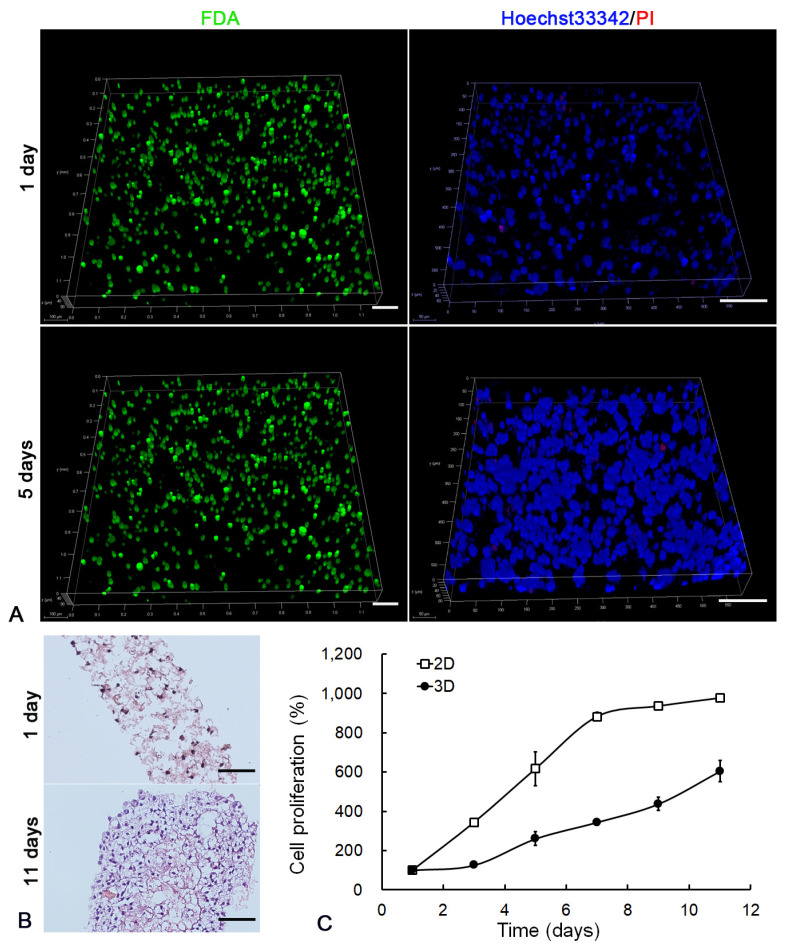
Cancer cell 3D cultured in LEH. (**A**) LSCM images of cancer cells in LEH at day 1 and 5. The morphology of cells embedded in the hydrogel was shown by FDA (green) staining. The live/dead assay was displayed by Hoechst 33342/PI (blue/red) staining. Scale bar: 100 μm. (**B**) H&E staining of 3D structure sections at time points of 1 and 11 days. Scale bar: 100 μm. (**C**) The proliferation of cancer cells in LEH was detected by MTT assay.

The lung cancer cell line A549 was utilized to examine the biocompatibility of LEH. FDA staining showed that cancer cells (green) uniformly dispersed in LEH on the first day and formed multi-cellular clusters after 5-day culture (Figure 3A). Hoechst33342/PI staining (blue/red) further demonstrated the formation of multi-cellular clusters (blue) with minimal apoptotic cells (red) in LEH (Figure 3A). H&E staining of 3D structure sections showed the homogenous distribution of cells on the first day and multilayer cellular structure after 11-day culture (Figure 3B). The growth curve indicated that cancer cells in LEH exhibited sustained proliferation at least to 11 days (Figure 3C). The 3D culture of cancer cells based on LEH was successfully developed after 11-day culture. 

### 2.3. Improvement of Metastatic Properties in 3D Cells

**Figure 4:**
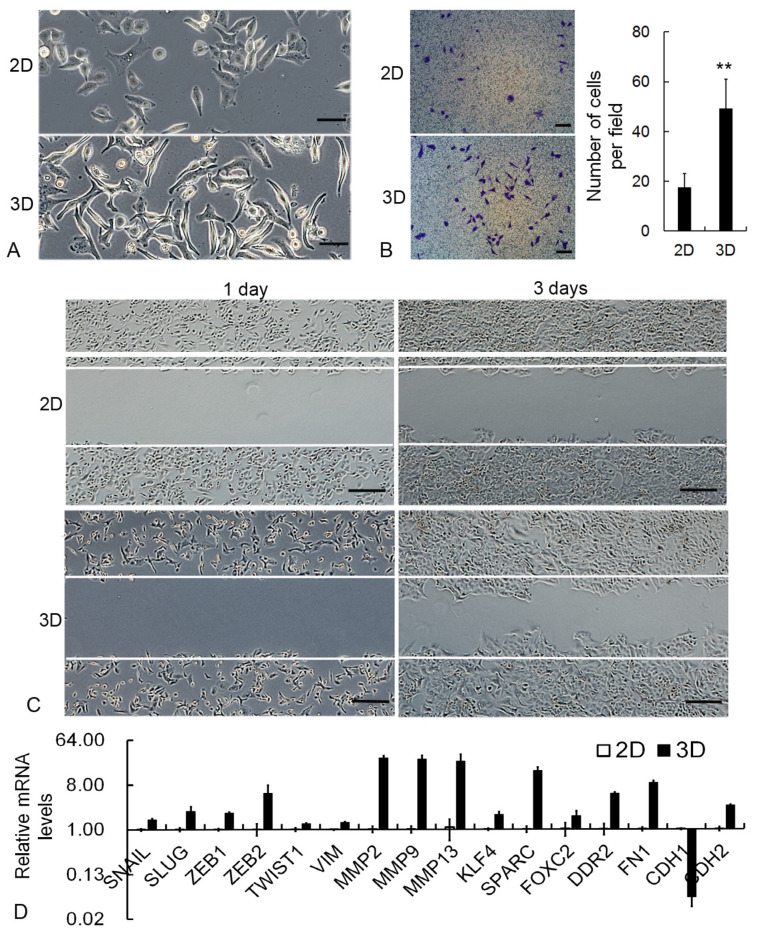
Upregulation of metastatic properties in 3D cancer cells. (**A**) Representative images of cancer cell morphological alternation. Scale bar: 50 μm. (**B**) Representative images and quantitative results of Trans-well migration assay. Scale bar: 100 μm. (**C**) Representative images of scratch wound healing assay. Scale bar: 200 μm. (**D**) The relative transcription level of metastasis-related genes as measured by qRT-PCR. GAPDH mRNA was utilized to normalize gene expression data, and the results were relative to 2D cells. ** *p* < 0.01.

In order to evaluate the influence of LEH-based 3D culture on cancer cell metastasis, Trans-well migration analysis, wound healing assay, and qRT-PCR were performed. After being transferred from 3D back to 2D condition, most cancer cells displayed morphological alternation from the epithelial to mesenchymal phenotype (Figure 4A). The number of 3D cells across the membrane was significantly higher than those from 2D in the Trans-well assay (Figure 4B). Compared with 2D cells, 3D cancer cells displayed increased motility in the scratch wound healing assay (Figure 4C). In accordance with the improved migration behavior of cell biology, the transcription level of metastasis-related genes, including *SNAIL*, *SLUG*, *ZEB1*, *ZEB2*, *TWIST1*, *VIM*, *MMP2*, *MMP9*, *MMP13*, *KLF-4*, *SPARC*, *FOXC2*, *DDR2*, *FN1*, and *CDH2*, was significantly improved in 3D (Figure 4D). However, the epithelial marker gene *CDH1* was downregulated in 3D cells. 

### 2.4. Upregulation of Self-Renewal in 3D Cells

**Figure 5:**
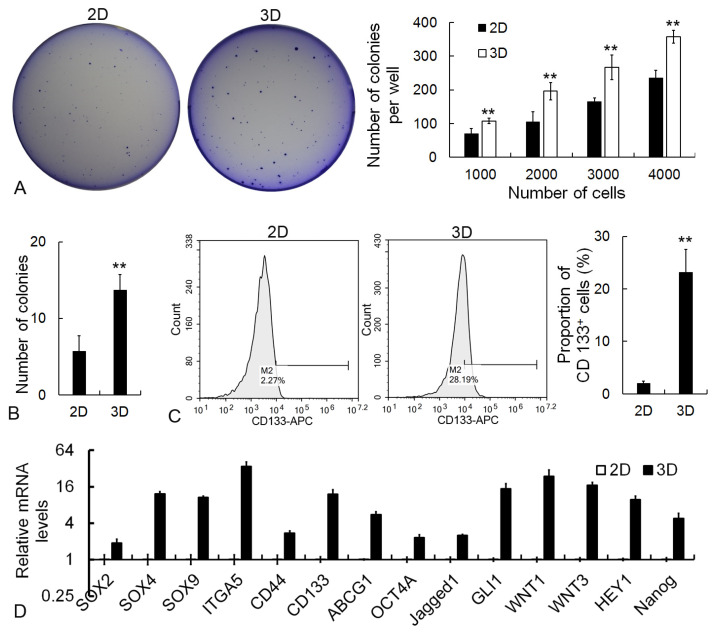
Analysis of self-renewal improvement in 3D cells. (**A**) Representative images and statistical results of soft agar colony formation assay. (**B**) Single-cell colony formation assay of cancer cells. (**C**) Flow cytometric analysis of CD133^+^ cells. (**D**) The relative transcription level of CSC-associated genes as measured by qRT-PCR. GAPDH mRNA was utilized to normalize gene expression data, and the results were relative to 2D cells. Scale bar: 100 μm. ** *p* < 0.01.

To detect the effect of 3D culture on CSC self-renewal properties, colony formation assay, flow cytometry analysis, and qRT-PCR were employed. The soft agar colony assay results displayed that when the same number of cells was adopted, 3D cancer cells could form a remarkably larger number of colonies than 2D cells (Figure 5A). The single-cell colony formation assay further confirmed the improved self-renewal capability of cancer cells under 3D conditions (Figure 5B). The flow cytometry assay showed that the percentage of cancer stem-like cells (CD133^+^) in the 3D condition was higher than that in 2D (Figure 5C). The expression level of CSC marker genes was detected by the qRT-PCR assay. As shown in Figure 4D, stemness-associated genes, including *SOX2*, *SOX4*, *SOX9*, *ITGA5*, *CD44*, *CD133*, *ABCG1*, *OCT4A*, *Jagged1*, *GLI1*, *WNT1*, *WNT3*, *HEY1*, and *Nanog*, were significantly upregulated in 3D cells (Figure 5D).

### 2.5. Cancer Cell Response to Anti-Cancer Drugs

**Figure 6:**
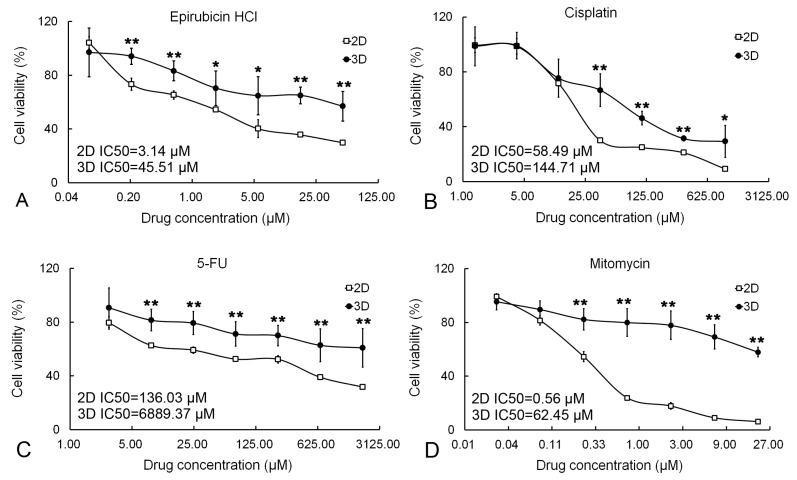
The drug resistance of cancer cells cultured in 2D and 3D. Anti-cancer drugs, including epirubicin HCl (**A**), cisplatin (**B**), 5-FU (**C**), and mitomycin (**D**), were utilized to treat cancer cells both in 2D and 3D conditions. Cell viability was determined by MTT assay. Results were shown as mean ± S.D. IC50 values for 2D and 3D cell cultures were given. * *p* < 0.01 and ** *p* < 0.01.

To examine the drug resistance of cancer cells in 2D and 3D conditions, clinical anti-cancer drugs, including epirubicin HCl, cisplatin, 5-FU, and mitomycin with different concentrations, were employed. The result showed that 3D cells displayed lower chemosensitivity to anti-cancer drugs than 2D cells (Figure 6). Accordingly, the IC50 values of epirubicin HCl, cisplatin, 5-FU, and mitomycin for 3D cells were 45.51 μM, 144.71 μM, 6889.37 μM, and 62.45 μM, while those for 2D cells were 3.14 μM, 58.49 μM, 136.03 μM, and 0.56 μM, respectively. 

### 2.6. Inhibitory Effects of EGCG on CSC Properties

**Figure 7:**
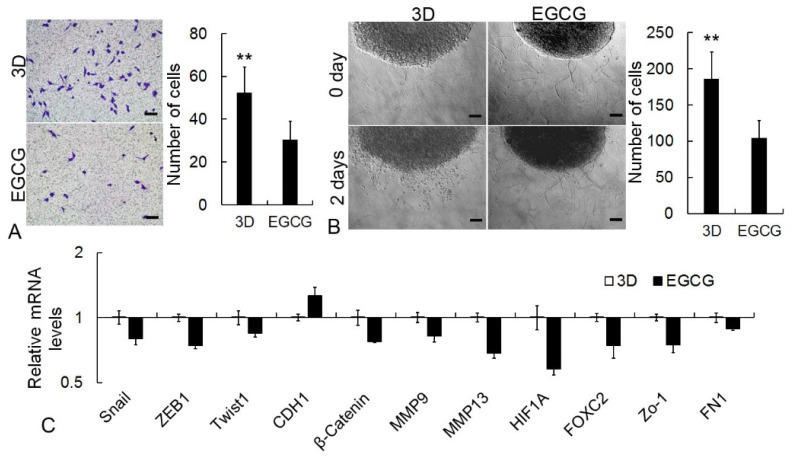
Inhibition effects of EGCG on CSC migration and invasion properties. (**A**) Trans-well assay. (**B**) Hydrogel invasion assay of 3D structure. The number of cells invading into hydrogel was calculated. Scale bar: 100 μm. (**C**) qRT-PCR assay of metastasis-related genes. GAPDH mRNA was utilized to normalize gene expression data, and the results were relative to 3D control. ** *p* < 0.01.

**Figure 8:**
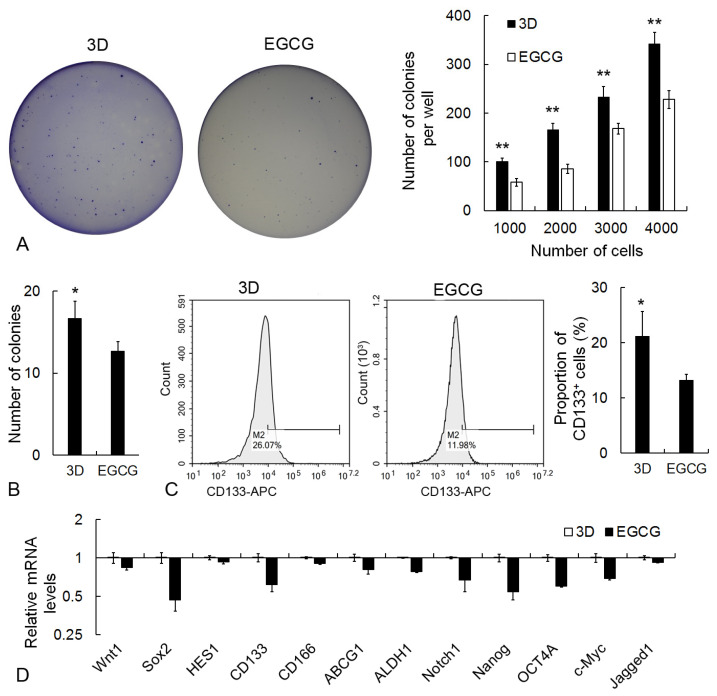
Attenuation effects of EGCG on CSC self-renewal properties in 3D condition. (**A**) Soft agar colony formation assay. (**B**) Single-cell colony formation assay. (**C**) Flow cytometry analysis of CD133^+^ proportion. (**D**) qRT-PCR assay of CSC-associated genes. GAPDH mRNA was utilized to normalize gene expression data, and the results were relative to 3D control. * *p* < 0.05 and ** *p* < 0.01.

EGCG was employed to examine the application of the 3D culture system on CSC-specific drug screening. Firstly, the migration and invasion properties of 3D cancer cells were analyzed after EGCG treatment. The migration of cancer cells was significantly attenuated by EGCG in the Trans-well assay (Figure 7A). Meanwhile, the invasive characteristics of 3D cancer cells were obviously inhibited by EGCG, as shown in the hydrogel invasion assay (Figure 7B). In addition, consistent with the results of cellular biological behaviors, the expression level of metastasis-associated genes, including *Snail*, *ZEB1*, *Twist1*, *β-Catenin*, *MMP9*, *MMP13*, *HIF1A*, *FOXC2*, *Zo-1*, and *FN1*, was significantly downregulated whereas the epithelial marker CDH1 was upregulated in EGCG-treated 3D cancer cells (Figure 7C). In the following detection, the effect of EGCG on self-renewal was identified in the 3D condition. EGCG significantly restrained the colony formation capability of 3D cancer cells (Figure 8A,B). The proportion of CD133^+^ stem-like cells was also reduced by EGCG in flow cytometry analysis (Figure 8C). Furthermore, the transcriptional level of CSC-associated genes, including *Wnt1*, *Sox2*, *HES1*, *CD133*, *CD166*, *ABCG1*, *ALDH1*, *Notch1*, *Nanog*, *OCT4A*, *c-Myc*, and *Jagged1*, was suppressed by EGCG (Figure 8D). 

## 3. Discussion

Hydrogel, with a spectrum of biophysical and biochemical properties, including designability in shape and topography, chemical modifiability, controllability in molecular structure, and the improvement of interactions between cell and materials, is a relatively ideal material for cancer cell culture [34,35,36]. Hydrogel-based 3D cancer models not only had a similar response to the treatment of clinic drugs as with individual patients but also offered effective platforms for the study of the interaction between cancer cells and microenvironments [37,38]. Therefore, various hydrogels from natural and synthetic materials were developed to design 3D cancer models for fundamental research and different types of therapies [39,40]. Considering the important role of ECM components on CSC metastasis, self-renewal, tumorigenicity, and drug resistance [41], the present study developed LEH to construct 3D cancer models. The main ECM components, including collagens, laminin, and fibronectin, were preserved in LEH. These ECM components provided special sites for cell attachment and proliferation via specific receptors and played an important role in the enrichment of growth factors and nutrients essential for cell survival, aggregation, and proliferation [42,43]. Lung cancer cells imbedded in LEH displayed a sustained proliferation state and formed multilayer cellular 3D architecture, confirming the excellent cytocompatibility of the LEH. 

Considering that the 3D culture system has been confirmed as an efficient way for CSC enrichment [44,45], the present study evaluated the improvement of CSC properties, including metastasis ability, self-renewal capacity, the proportion of cancer stem-like cells, and chemoresistance in 3D cancer cells. Lung cancer cells in LEH exhibited enhanced metastasis with an overexpression of metastasis-related genes. These results were similar to the previous study in which cancer cells in the 3D condition displayed the improvement of migration and invasion properties, thus providing a predictive strategy for drug discovery of antimetastatic therapeutics [46,47]. Besides the enhanced metastasis, the self-renewal capability of cancer cells was also significantly improved in 3D by upregulating stemness-related genes. Notably, the proportion of CSC-like cells (CD133^+^) in 3D was higher than that in 2D, confirming the enrichment of CSCs in LEH-based 3D conditions. Apart from the improved cell–cell interaction, hypoxic microenvironment, and chemical gradients, the ECM microenvironments, including components, structure, and physical and chemical properties, also play dominant roles in the enhanced CSC properties [48,49]. For example, ECM protein fibronectin could activate Wnt/β-catenin pathway to induce the stem cell phenotype in cancer cells by binding membrane receptor integrin β1, while a disruption of this interaction could inhibit CSC self-renewal and tumor-initiating capacity [50]. Notably, the conversion from differentiated cancer cells to highly tumorigenic CSC-like cells, induced by ECM components around cancer cells, might be attributed to the enrichment of CSCs in 3D conditions [51]. With the exceeding self-renewal property of 3D cancer cells, the good performance in drug resistance was detected. The result was consistent with previous studies that cancer cells cultured in 3D conditions displayed enhanced drug tolerance [52,53]. The improved drug resistance of 3D cancer cells may be partially attributed to the physical barriers by enhanced cell–cell and cell–ECM interactions. The more potentially important reason was the overexpression of CSC-associated genes [54]. 

Following the detection of the improved CSC properties, the practicability of the 3D cancer model in screening CSC-specific drugs was examined by testing the pharmacological effect of EGCG on CSC properties. EGCG, an important functional component of polyphenol in green tea, has potent anti-cancer effects by inhibiting cell proliferation, suppressing angiogenesis, inducing apoptosis, and enhancing anti-tumor immunity in various cancers [55,56]. In addition, EGCG displays the inhibition effect on CSCs’ properties by the attenuation of migration, invasion, self-renewal, and therapeutic resistance in 2D conditions [57,58]. Firstly, the present study found that EGCG could attenuate the diffusion rate of cancer cells from the 3D structure to the microenvironment by downregulating metastasis-associated genes. A simple and convenient in vitro 3D model for screening new pharmacological drug candidates on cancer cell metastasis was constructed in this progress. Secondly, the inhibitory effect of EGCG on self-renewal properties in the 3D model was also demonstrated by downregulating CSC-associated genes, restraining colony formation capability, and reducing CSC proportion. Our results were similar to the previous studies in which EGCG inhibited cancer cells’ stemness in 3D conditions [59,60,61,62]. The attenuation of stemness-associated signal pathways, including STAT3, Wnt/β-catenin, Notch, and sonic hedgehog signaling, may be involved in this progress [63,64]. Overall, the inhibition effect of EGCG on CSC properties in the 3D condition confirmed the practicability of LEH in CSC-target drug screening. 

## 4. Materials and Methods

### 4.1. Materials

Fresh porcine lung tissues were purchased from the Xinyang Slaughter market. Sodium dodecyl sulfate, Triton X-100, DNase I, Fluorescein diacetate (FDA), Hoechst33342/PI, pepsin solution, and paraformaldehyde were purchased from Sigma-Aldrich Co., Ltd., Burlington, MA, USA. Hematoxylin and eosin (H&E), Masson’s Trichrome (MT), Alcian Blue (AB) and Elastin Van Gieson (EVG), DNA isolation kit, and (4,5-dimethylthiazole-2-yl 2,5-diphenyl tetrazolium bromide) (MTT) solution were obtained from Solarbio Co., Ltd., Beijing, China. Diaminobenzidine (DAB substrate kit) was obtained from ZSGB-BIO Co., Ltd., Beijing, China. Millipore water was obtained from the equipment Milli-Q50 SP (Millipore Corporation, St. Louis, MO, USA). All chemicals were employed without further treatments. 

### 4.2. Preparation of Lung dECM Hydrogel (LEH) 

The decellularized lung matrix was prepared according to the previous reports, and the method was modified [65,66]. First, the fresh porcine lung tissues were freeze–thaw-treated 3 times, and the tissues were chopped into about 3 mm thickness and directly washed with 1% sodium dodecyl sulfate solved in Millipore water at 37 °C for 3 days. The decellularization solution was regularly changed every 6 h. Following this, the matrix was washed with 1% Triton X-100 solution for 12 h. And then, the materials were washed with Millipore water for 2 days and put into 0.5 mg/mL DNase I solutions for 1 h. Lastly, the materials were immersed in 1 mg/mL pepsin solution to obtain the decellularized lung matrix. Lyophilized ECM sample was mixed into 0.5M acetic acid at a ratio of 20 mg/mL and constantly agitated for 48 h at low temperature (less than 5 °C). After being centrifuged at 1000× *g* for 20 min, the supernatant of the mixture was freeze-dried overnight. For the preparation of lung dECM hydrogel (LEH), 10 mg/mL lyophilized ECM scaffold was added into 0.1M acetic acid with continuous stirring for 24 h and neutralized to pH 7.2 with 1M NaOH on ice. With the increase in temperature over 25 °C, LEH was obtained. All the materials were sterilized by Colbat-60. 

### 4.3. Rheological Properties of LEH 

A rheometer (Waters^TM^, New Castle, TA, USA) was used to evaluate the sol–gel transition behavior and the rheological properties of LEH [32]. The parallel plate geometry using 40 mm diameter was used, the storage modulus (G′) and loss modulus (G″) were recorded over time using 1% strain and 1 Hz frequency to monitor the hydrogel formation process. Before the test, the cone was lowered to 1 mm gap, and the silicone oil was used to enclose the outside edge of the cone for preventing the evaporation of water.

### 4.4. Microstructure of LEH 

A field emission scanning electron microscope (Hitachi S-4800, Hitachi, Japan) was used to observe the microstructure of LEH. First, LEH was freeze-dried; then, the lyophilized sample was immersed in liquid nitrogen for 2 min and broken, and the fracture surface was sprayed with platinum (40 s sputter). Then, SEM images of LEH were generated, and the pore size distribution was determined based on five SEM images using ImageJ software (Version 1.54) analysis.

### 4.5. Histological and Immunohistochemical (IHC) Staining

The decellularized samples were fixed with 4% paraformaldehyde and embedded in paraffin for sections by microtome (Microscopy GmbH 2.3, Carl Zeiss, Gottingen, Germany). After being deparaffinized and rehydrated, slides were stained with Hematoxylin and eosin (H&E), Masson’s Trichrome (MT, for collagen), Alcian Blue (AB, for glycosaminoglycans) and Elastin Van Gieson (EVG, for elastin) according to the introduction. IHC staining was performed following the manufacturer’s instructions. The slides were immersed in 1% H_2_O_2_ for 20 min and in normal goat serum for 1 h. Primary antibodies against fibronectin, laminin, collagen I, collagen III, and collagen IV (Abcam) were added overnight at 4 °C. Then, sections were developed with diaminobenzidine (DAB substrate kit) staining. The section without the primary antibody was used as control. 

### 4.6. Quantification of DNA Content

The DNA content of fresh lung tissue and LEH was quantified using a DNA isolation kit according to the manufacturer’s protocol. Briefly, the samples were digested by Proteinase K for 8 h. DNA samples were purified using alcohol washes and quantified using a NanoDrop 2000 spectrophotometer (Thermo Fisher Scientific 4.0.1, Waltham, MA, USA) at 260/280 nm ratio. 

### 4.7. Three-Dimensional Cell Culture in LEH

Established human lung cancer cell line A549 was purchased from Cell Resource Center, IBMS, CAMS/PUMC (Beijing, China) and cultured in RPMI-1640 medium supplemented with 10% fetal bovine serum (FBS), 1.0 mM sodium pyruvate, and 0.1 mM non-essential amino acids (all from Gibco; Thermo Fisher Scientific, Waltham, MA, USA) in a humidified environment at 37 °C with 5% CO_2_. Cells were passaged every 2–3 days by 0.25% trypsin (Thermo Fisher Scientific). For 3D structure formation, 1 × 10^4^ cells were imbedded in 5 μL pre-hydrogel at a lower temperature (around 10 °C); with increasing temperature over 30 °C, the cell-laden LEH was obtained. The cell-capsulated LEHs were cultured in the glass plate under a humidified environment (37 °C) with 5% CO_2_. The cell culture medium was changed every day. For cell detachment, the 3D structures were washed with D-PBS 3 times, incubated in 0.25% trypsin at 37 °C for 10 min on shaker, and manually pipetted in a cell culture medium. Cells were centrifuged at 250× *g* and re-suspended in fresh cell culture media for utilization. Trypan blue staining was utilized to distinguish between viable and nonviable cells.

### 4.8. Cytocompatibility Analysis of LEH 

Fluorescein diacetate (FDA) and Hoechst33342/PI staining were performed to assay the cytocompatibility of LEH. The 3D structures were immersed into FDA solution for 2 min and washed with D-PBS for FDA staining. The images of cells were captured by laser-scanning confocal microscopy (LSCM, Leica Microsystems CMS GmbH, Leica TCS SP8, Wetzlar, Germany). For Hoechst33342/PI staining, 3D cells were immersed in Hoechst33342 solution for 30 min in darkness. After being washed with D-PBS for 3 times, the 3D structures were stained with PI for 3 min and imaged by LSCM. 

### 4.9. Cell Viability Assays in LEH

Cellular proliferation and drug cytotoxicity were determined using the 3-(4,5-dimethylthiazole-2-yl 2,5-diphenyl tetrazolium bromide) (MTT) assay. Mainly, cells were immersed in a cell culture medium with 0.5 mg/mL MTT for 6 h. DMSO with 100 μL was adopted to solve the purple crystal on an orbital incubator at 37 °C for 15 min. Enspire^®^ Multimode Plate Reader (PerkinElmer Life Sciences, Waltham, MA, USA) was utilized to measure the solution at a wavelength of 570 nm. The IC50 (50% inhibition) values of drugs were calculated.

### 4.10. Gene Expression Analysis

Total RNA was extracted from cells using TRIzol solution (Invitrogen, Waltham, MA, USA) by the manufacturer’s protocol and quantified by Nanodrop 2000. cDNA was synthesized from 2 μg total RNA using random primers and a Transcriptor First Strand cDNA Synthesis kit (Roche Applied Science, Penzberg, Germany). Quantitative RT-PCR (qRT-PCR) analysis was performed in a Roche LightCycler480 with 45 cycles, each consisting of 10 s at 95 °C, 15 s at 60 °C, and 20 s at 72 °C followed by a melting curve analysis, using the Takara SYBR^®^ Premix Ex Taq^TM^. Relative gene expression was calculated as the ratio to GAPDH using the 2^−ΔΔCt^ method.

### 4.11. Cell Migration and Invasion Assay

The migration and invasion ability of cancer cells were displayed by Trans-well, scratch wound healing, and hydrogel invasion assay. For the Trans-well assay, 1 × 10^4^ cells in 200 μL medium without FBS were plated on upper chambers with 8 μm pore size (Corning, Corning, NY, USA). After 48 h, cells on the upper surface of the chambers were removed, while cells at the bottom were stained with 0.5% crystal violet. The images of cells were obtained by microscope (Zeiss GmbH, Jena, Germany). The number of cells across 8 μm pore was quantified. For the scratch wound healing assay, cells were seeded in a 6-well plate with 50–60% confluent and scratched. Cell migration to absent space was imaged by microscope after 2 days. For hydrogel invasion assay, cold 10 mg/mL LEH was placed on 24-well plates with 300 μL per well, and the 3D cultures were immediately immersed. The 24-well plate was inversely placed in CO_2_ incubation at 37 °C for at least 30min. Then, a fresh cell culture medium was added into the well. Cell invasion into LEH was imaged by the microscope, and the number was quantitatively analyzed. 

### 4.12. Colony Formation Assay

Single-cell and soft agar colony formation assays were carried out to detect the self-renewal properties of cancer cells. For single-cell colony formation assay, a single cell in 120 μL medium was seeded in Coster ^®^ ultra-low attachment 96-well plate (Corning). Colonies were directly calculated in a microscope after 12 days of incubation. For soft agar colony formation assay, 0.6% agar in 1 mL cell culture medium was added to 6-well plates for the base layer. Cancer cells with 1000, 2000, 3000, and 4000 in 0.35% agar solution were added as the top layer. After 20 days of incubation, colonies were stained with 0.2% crystal violet (Sigma-Aldrich) in 95% ethanol for 10 min. Colonies were photographed and quantified. 

### 4.13. Flow Cytometry Analysis

Single-cell suspension in D-PBS with 2% FBS was prepared. Cell solution with a concentration of 10^6^/mL were incubated with 5 μL anti-human CD133-APC (eBioscience, San Diego, CA, USA) or mouse IgG-APC on ice in dark for 30 min according to the manufacturer’s protocol. Cells were washed 3 times with D-PBS and re-suspended in D-PBS in darkness. The proportion of CD133^+^ cells was evaluated with a NovoCyte Flow Cytometer (ACEA Biosciences 1.2.4, San Diego, CA, USA).

### 4.14. Statistical Analysis 

All results were expressed as mean ± standard deviation (S.D.). Differences between the two groups were analyzed with *t*-tests using a SPSS 19.0 software (SPSS Inc., Chicago, IL, USA). Statistical significances were considered as * *p* < 0.05 and ** *p* < 0.01.

## 5. Conclusions

In summary, we have developed a thermo-responsive hydrogel based on porcine lung decellularized ECM for a 3D cell culture system to explore CSC characteristics and CSC-target drug screening. It was observed that the main CSC properties, including improved metastasis, CSC proportion, self-renewal, and drug tolerance, could be upregulated in this 3D cell culture system. Significantly, the utilization of the obtained hydrogel-based 3D culture in CSC-target drug screening was characterized by examining the inhibition effects of EGCG on CSC properties. Although it is acknowledged that the results in the present study are preliminary, the LEH-based 3D culture system presents a foundation for further investigations in biological, pharmacological, and toxicological fields, especially providing a simple and effective in vitro model for the discovery of new CSC-specific drugs. 

## Data Availability

Data are contained within the article.
